# Agarooligosaccharides as a novel concept in prebiotics: selective inhibition of Ruminococcus gnavus and Fusobacterium nucleatum while preserving Bifidobacteria, Lactobacillales in vitro, and inhibiting Lachnospiraceae in vivo

**DOI:** 10.1099/mic.0.001510

**Published:** 2024-11-21

**Authors:** Tadashi Fujii, Koji Karasawa, Hideaki Takahashi, Ikuya Shirai, Kohei Funasaka, Eizaburo Ohno, Yoshiki Hirooka, Takumi Tochio

**Affiliations:** 1 Department of Gastroenterology and Hepatology, Fujita Health University, Toyoake, Aichi, Japan; 2 Department of Medical Research on Prebiotics and Probiotics, Fujita Health University, Toyoake, Aichi, Japan; 3 BIOSIS Lab. Co., Ltd., Toyoake, Aichi, Japan; 4 Research & Development, Ina Food Industry, Co., Ltd., Ina, Nagano, Japan; 5 Graduate School of Nutritional Sciences, Nagoya University of Arts and Sciences, Nisshin, Aichi, Japan

**Keywords:** agarooligosaccharides, Bifidobacteria, *fab*, *Fusobacterium nucleatum*, Lactobacillales, *Ruminococcus gnavus*

## Abstract

Recent studies have linked *Ruminococcus gnavus* to inflammatory bowel disease and *Fusobacterium nucleatum* to various cancers. Agarooligosaccharides (AOS), derived from the acid hydrolysis of agar, have shown significant inhibitory effects on the growth of *R. gnavus* and *F. nucleatum* at concentrations of 0.1 and 0.2%, respectively. RNA sequencing and quantitative reverse-transcription PCR analyses revealed the downregulation of fatty acid biosynthesis genes (*fab* genes) in these bacteria when exposed to 0.1% AOS. Furthermore, AOS treatment altered the fatty acid composition of *R. gnavus* cell membranes, increasing medium-chain saturated fatty acids (C8, C10) and C18 fatty acids while reducing long-chain fatty acids (C14, C16). In contrast, no significant growth inhibition was observed in several strains of Bifidobacteria and Lactobacillales at AOS concentrations of 0.2 and 2%, respectively. Co-culture experiments with *R. gnavus* and *Bifidobacterium longum* in 0.2% AOS resulted in *B. longum* dominating the population, constituting over 96% post-incubation. *In vivo* studies using mice demonstrated a significant reduction in the Lachnospiraceae family, to which *R. gnavus* belongs, following AOS administration. Quantitative PCR also showed lower levels of the *nan* gene, potentially associated with immune disorders, in the AOS group. These findings suggest that AOS may introduce a novel concept in prebiotics by selectively inhibiting potentially pathogenic bacteria while preserving beneficial bacteria such as Bifidobacteria and Lactobacillales.

**Impact Statement**

This study advances the introduction of a novel concept in prebiotics by demonstrating the unique effects of agarooligosaccharides (AOS) on the microbiome. By specifically inhibiting the growth of disease-associated bacteria such as *Ruminococcus gnavus* and *Fusobacterium nucleatum* – implicated in inflammatory bowel disease and cancer, respectively – while not affecting beneficial bacteria like Bifidobacteria and Lactobacillales, AOS offers a targeted approach to microbiome modulation. Furthermore, our research indicates that AOS significantly downregulates the transcription of the fatty acid biosynthesis genes in the pathogenic bacteria studied. The broad interest of this research lies in its utility for developing microbiome-based interventions that could improve human health without compromising beneficial microbial populations. The findings represent a significant advancement in the novel concept of prebiotic research, offering potential implications for dietary strategies and therapeutic interventions targeting microbial-associated diseases. This contribution is highly relevant to microbiome researchers, clinical practitioners and the broader community interested in non-pharmacological approaches to disease management.

## Introduction

Maintaining a healthy balance in the gut microbiota is crucial for overall well-being, given its vital role in various physiological processes and immune system regulation [1]. Dysbiosis, characterized by an imbalance in gut microbial composition, can foster the proliferation of harmful bacteria and contribute to gastrointestinal disorders [1]. This has led to increasing interest in natural compounds that not only support the growth of beneficial bacteria but also inhibit pathogenic bacteria in the gut [2]. Prebiotics, in particular, have gained significant attention for their health benefits. The concept of prebiotics was first introduced in 1995 by Glenn Gibson and Marcel Roberfroid [3]. They defined prebiotics as ‘non-digestible food ingredients that beneficially affect the host by selectively stimulating the growth and/or activity of specific bacteria in the colon, thereby improving host health’. In 2008, at the sixth Meeting of the International Scientific Association of Probiotics and Prebiotics (ISAPP), the term ‘dietary prebiotics’ was redefined as ‘a selectively fermented ingredient that leads to specific changes in the composition and/or activity of the gastrointestinal microbiota, thus providing health benefits to the host’ [4].

Agar, a natural polysaccharide sourced from the cell walls of red algae, is the precursor to agarooligosaccharides (AOS). These low-molecular-weight sugars, derived from agar, hold significant potential for applications across the food, cosmetic and pharmaceutical industries due to their diverse physiological activities, including prebiotic, antioxidant, anti-inflammatory and hepatoprotective effects [5,6]. Agarooligosaccharides are categorized into two types: AOS and neoagarooligosaccharide (NAOS), characterized by *α*-1,3-linked-3,6-anhydro-l-galactose (AHG) and *β*-1,4-linked-d-galactose at their reducing ends, respectively [6]. AOS are produced industrially through the acid hydrolysis of agar, with the degree of polymerization (DP) varying based on the conditions of preparation [5,6].

A study on the prebiotic activity of AOS *in vitro* fermentation by human faecal inocula reported that AOS can increase the concentrations of short-chain fatty acids and modulate the composition of the microbiota [7]. Recently, AHG was reported to show growth-inhibitory activity against *Streptococcus mutans* [8], an acid-producing bacterium considered a primary pathogen associated with dental caries [9]. Moreover, AOS and agarobiose, a DP2 unit of AOS carrying AHG at the reducing ends, also showed growth-inhibitory activity against *S. mutans*, while NAOS did not [10].

*Ruminococcus gnavus*, a Gram-positive bacterium, colonizes the intestinal mucosa and utilizes sialic acid from mucin sugar chains as a carbon source. This process is facilitated by sialidase activity, driven by proteins encoded within its *nan* gene cluster [11]. This metabolic pathway may play a role in disrupting the gut barrier, potentially contributing to a leaky gut and associated immune disorders. In healthy individuals, the average relative abundance of *R. gnavus* in the intestine is about 0.1%, but this can rise to as much as 69% in patients with inflammatory bowel disease (IBD) [12]. *R. gnavus* is known for producing inflammatory polysaccharides, particularly glucorhamnan, which activates dendritic cells through toll-like receptor 4 on the host. This activation triggers the release of inflammatory cytokines, such as TNF-α and Il-6, potentially leading to the onset of Crohn’s disease, a type of IBD [13]. In a previous quantitative PCR study, we detected elevated intestinal levels of the *nan* gene in various immune-related conditions, including allergies in mice and ulcerative colitis, another form of IBD, in humans [14]. *R. gnavus* has undergone several taxonomic revisions over time. Initially classified within the genus *Ruminococcus*, it was more recently reclassified into the genus *Mediterraneibacter* belonging to Lachnospiraceae family [15]. To maintain consistency, this paper will refer to it as *R. gnavus* throughout.

*Fusobacterium nucleatum*, a Gram-negative bacterium, is prominently found in colorectal carcinoma tissues [16,17]. It interacts with colorectal cancer cells by adhering to and invading them, utilizing its unique FadA adhesin to induce oncogenic and inflammatory responses that promote tumour growth [9]. Additionally, *F. nucleatum*’s outer membrane proteins, Fap2 and RadD, stimulate the host to produce inflammatory factors and recruit inflammatory cells, thereby fostering a tumour-supportive environment [18].

In contrast, Bifidobacteria and Lactobacillales offer numerous health benefits, including antimicrobial and immunomodulatory effects [19,22]. Their protective mechanisms involve anti-adhesion factors and the production of pathogen-inhibiting by-products such as hydrogen peroxide, bacteriocins and organic acids like acetic acid and lactic acid, which help suppress pathogenic microbes. Additionally, they play a role in modulating immune responses and signalling pathways.

Prebiotics are often short-chain carbohydrates that selectively boost the activity of specific beneficial bacteria [23]. In this study, we present AOS as a novel concept in prebiotics – demonstrating its ability to selectively inhibit the growth of potentially pathogenic bacteria, such as *R. gnavus* and *F. nucleatum*, while allowing beneficial bacteria like Bifidobacteria and Lactobacillales to thrive at the same concentrations. Through AOS exposure assays and transcriptomic analysis, we demonstrate that this growth inhibition would be driven by the suppression of fatty acid biosynthesis gene expression in both *R. gnavus* and *F. nucleatum*. Co-culture assays with AOS further emphasize its beneficial effects, promoting the growth of beneficial *Bifidobacterium longum* over pathogenic *R. gnavus* in the media. Moreover, *in vivo* studies using mice showed a significant reduction in the relative abundance of the Lachnospiraceae family, to which *R. gnavus* belongs, along with a significant decrease in the levels of its potentially pathogenic *nan* gene following AOS intervention.

## Methods

### Bacterial strain and preparation of media

The type strains *R. gnavus* JCM 6515, *F. nucleatum* JCM 8532, *B. longum* subsp. *longum* JCM 1217, *Bifidobacterium adolescentis* JCM 7046 and *Lacticaseibacillus casei* JCM 1134 were obtained from the Japan Collection of Microorganisms (JCM). *Lactiplantibacillus plantarum* FM8 was supplied by MINORI Inc. (Tokyo, Japan). For cultivation, except for Lactobacillales (*L. plantarum* and *L. casei*), we used the Reinforced Flora growth (RF) medium, which is a modified growth medium described in the literature [24]. Briefly, RF medium is a brain heart infusion broth (Thermo Fisher Scientific, Waltham, MA, USA) supplemented with 5 g yeast extract (Becton Dickinson, Sunnyvale, CA, USA) per litre, 5 g K_2_HPO_4_ (Fujifilm-Wako, Osaka, Japan), 8 g glucose (Fujifilm-Wako), 0.5 g l-cysteine-hydrochloride (Sigma Aldrich, St. Louis, MO, USA), 1 g Tween 80 (Tokyo Kasei Chemical Company, Tokyo, Japan), 0.005 g hemin (Fujifilm Wako), 0.002 g vitamin K1 (Fujifilm Wako), 0.001 g resazurin sodium salt (Tokyo Kasei Chemical Company), 0.025 g acetate (Fujifilm Wako) and 0.01 g MgSO_2_7H_2_O (Fujifilm-Wako) (pH 6.8). In some cases, 20% glucose (Fujifilm-Wako, Osaka, Japan) or 10% AOS (or its fractionated sugars) solutions that were filtered through a cellulose acetate filter (0.2 µm, DISMIC-25CS, Advantec, Tokyo, Japan) were added to the RF medium prior to incubation. Lactobacillales were cultivated in de Man, Rogosa and Sharpe (MRS) broth (Merck-Millipore, Darmstadt, Germany). AOS was provided by Ina Food Industry Co., Ltd., whose composition of biose, tetraose, hexaose and octaose was 41.8, 41.0, 14.5 and 2.7% [25].

### Fractionation of AOS constituent sugars

Recycling size-exclusion chromatography was performed using a LaboACE LC-7080 Plus (Japan Analytical Industry Co., Ltd., Japan) equipped with JAIGEL-W252/W253 columns [mobile phase: 0.005% (v/v) acetic acid in 10% (v/v) ethanol; flow rate: 3.5 ml min^−1^] to separate the variously sized AOS, biose, tetraose, hexaose or octaose. Analytical HPLC data were recorded on a Nexera GPC system (Shimadzu Corporation, Japan) equipped with two tandems TSKgel G2500PWXL columns (300×7.8 mm I.D., Tosoh Corporation, Japan).

### Growth tests of *R. gnavus* and *F. nucleatum* with AOS and each fractionated constituent sugar of AOS

Strains of *R. gnavus* and *F. nucleatum* were each inoculated into RF medium containing 1% glucose and cultured at 37 °C for 23 h using the AnaeroPack system (Mitsubishi Gas Chemical Company Inc., Tokyo, Japan) to serve as an inoculum. After dispensing 470 µl of RF medium and 2.5 µl of 20% glucose containing each concentration of AOS (0, 0.1 and 0.2%) into 96-deep well plates (AxyGen Scientific, CA, USA), each well was inoculated with 25 µl of each bacterium and cultured at 37 °C using AnaeroPack system (*N* =8). AOS was added to a 10% aqueous solution. After incubation for 23 h (for *R. gnavus*) and 9 h (for *F. nucleatum*), 20 µl of these cultures was suspended in 180 µl of water in the 96-well flat-bottomed plates (4845-96F; Watson Bio Lab, CA, USA), and turbidity (OD_660_) was measured using a microplate reader (SpectraMax M2; Molecular Devices, CA, USA). Similarly, the growth inhibitory effects of 0.1% AOS and its component sugars (biose, tetraose, hexaose and octaose) on *R. gnavus* and *F. nucleatum* were tested after incubation for 23 and 41 h, respectively (*N* = 2). Statistical analyses were performed using the Kruskal–Wallis test for intragroup statistics followed by the *post-hoc* Dunn’s multiple comparisons test.

### RNA sequencing analysis

*R. gnavus* was inoculated into 3 ml of RF medium containing 0.1% glucose with or without 0.1% AOS in 15 ml polypropylene tubes (CELLSTAR® CELLreactor™ Greiner, Germany) (*N* = 3) and cultured at 37 °C using the AnaeroPack system. Samples containing AOS were incubated for 72 h with 100 µl of inoculum (final OD_660_ = 0.04), and samples without AOS were incubated for 48 h with 10 µl of inoculum (final OD_660_ = 0.21). After incubation, the cells were ice-cooled for 10 min and centrifuged at 6 000 r.p.m. for 5 min at 4 °C, and 0.6 ml of RNA later (Thermo Fisher Scientific) was added to the precipitate. RNA extraction and RNA sequencing (RNA-seq) were performed for three independent cultures under each condition according to procedures described previously [26]. RNAseq analysis and visualization, including PCA and volcano plots, were conducted using the iDEP.96 (http://bioinformatics.sdstate.edu/idep96/) with default settings. Differential expression analysis was performed using DESeq2, with *P*-value correction applied via the Benjamini–Hochberg [false discovery rate (FDR)] method. To ensure reproducibility, all parameters were maintained as per the default configuration in iDEP.96. The top 20 up- and downregulated genes in response to AOS obtained by RNA-seq analysis were annotated using protein analysis with *Z*-score (PANNZER2; http://ekhidna2.biocenter.helsinki.fi/sanspanz/) to predict the functional description (DE) and Gene Ontology (GO) classes of proteins.

### Quantitative reverse transcription PCR (RT-qPCR) analysis

The RT-qPCR analysis of *R. gnavus* and *F. nucleatum* was performed as follows. Total RNA of *R. gnavus* was prepared as mentioned in the section on ‘RNA sequencing analysis’. Total RNA from *F. nucleatum* was also prepared in the same manner (*N*=3), but samples containing AOS were incubated for 54 h with 80 µl of inoculum (final OD_660_=0.11), and samples without AOS were incubated for 24 h with 10 µl of inoculum (final OD_660_=0.125). Total RNA was extracted as described previously [27]. cDNA was synthesized using the Transcriptor First Strand cDNA Synthesis Kit (Roche Diagnostics, Ottweiler, Germany) with random primers and total RNA. Using Primer-blast (https://www.ncbi.nlm.nih.gov/tools/primer-blast/), primer sets were designed to detect *fabF* and *fabH* in *R. gnavus* and their homologous genes in *F. nucleatum* (Table 1).

**Table 1.** Primers used in the present study

Target gene	Oligonucleotide sequence	Annealing temperature (℃)	Reference	Used for
*R. gnavus gyrB*	GGAGCAGACCAGATCCAAAT	55	[11]	RT-qPCR and qPCR
	CCAATATACATTCCCGGTCTTT			
*R. gnavus fabF*	CAGAAAGCTGTATCTGCCCA	55	This study	RT-qPCR
	CCCTCCCCTAACACAAATCC			
*R. gnavus fabH*	AAAGGCAGCGTACATTCAGA	55	This study	RT-qPCR
	GATCCTTCTGTTCGCCTGAT			
*F. nucleatum* 16S rRNA gene	CGGGACTTAACCCAACATCT	58	[29]	RT-qPCR
	AGGAACCTTACCAGCGTTTG			
*F. nucleatum fabF*	TGCTCATGGAACTTCAACTCCT	58	This study	RT-qPCR
	CCATGTCCAGTTGCTCCCTT			
*F. nucleatum fabH*	ACTTCTGATTTGGCAACTGAAGC	58	This study	RT-qPCR
	AACTATACAAGCTGCCCCCTG			
*Bifidobacterium* 16S rRNA gene	GATTCTGGCTCAGGATGAACGC	55	[22]	qPCR
	CTGATAGGACGCGACCCCAT			
*nanA*	ATYCCGGCATTTTATGC	50	[14]	qPCR
	CCRTTTACRTAGACACCYTTTAC			
Total 16S rRNA gene	CGGTGAATACGTTCCCGG	60	[30]	qPCR
	TACGGCTACCTTGTTACGACTT			

RT-qPCR was performed using the QuantStudio 3 (Thermo Fisher Scientific). The reaction solution was prepared for each cDNA and primer set using the PowerTrack SYBR Green Master Mix (Thermo Fisher Scientific) according to the manufacturer’s instructions. The amplification cycle protocol consisted of an initial denaturation at 95 °C for 2 min, followed by 40 cycles of denaturation at 95 °C for 10 s, annealing at the temperature shown in Table 1 for 15 s, extension at 72 °C for 15 s and a final extension at 72 °C for 1 min. After amplification, a melting curve analysis was performed to confirm the specificity of the PCR products. Target mRNA levels were quantified using the ΔΔCt method [28] and normalized to the expression level of the reference gene *gyrB* for *R. gnavus* [11] or 16S rRNA gene for *F. nucleatum* [29]. Statistical analyses were performed using unpaired t-test.

### Fatty acid composition analysis in *R. gnavus* membrane

Cells for fatty acid composition analysis of *R. gnavus* membranes were prepared in the same manner as cells for RNA-seq (*N*=3). The lyophilized cells of *R. gnavus*, weighing 7 ± 2 mg, were transferred into a 4 ml vial and used as the sample. Methyl esterification of the fatty acids and purification of the methylated fatty acids were conducted using the Nacalai Tesque (Kyoto, Japan) fatty acid methylation kit (P/N: 06482-04) and the methylated fatty acid purification kit (P/N:06483–94). Of the 3 ml methyl-derivatized fatty acid sample, 1 ml was transferred to a vial for measurement and used as the GC–MS sample. The GC–MS analytical conditions are listed in Table 2. Statistical analyses were performed using the two-way ANOVA.

**Table 2.** GC–MS analytical conditions

Item	Conditions
**Instrument**	GC–MS-TQ8040 NX (Shimadzu), AOC-6000 (Auto injector, Shimadzu)
**GC conditions**	
**Column**	DB-5MS (30 m × 0.25 mm I.D., df = 0.25 µm; Agilent Technologies)
**Injection temperature**	250 °C
**Column temperature**	60 ℃ (1.0 min, 40 ℃ min^−1^)–200 ℃ (3.0 min, 25 ℃ min^−1^)–250 ℃ (5.5 min); total 15 min.
**Injection mode**	Split
**Carrier gas**	He (constant linear velocity)
**Linear velocity**	53.4 cm s^−1^
**Injection volume**	2 µl
**MS conditions**	
**Ion source temperature**	230 °C
**Interface temperature**	250 °C
**Measurement range**	*m*/*z* 35–600
**Event time**	0.3 s
**Scan speed**	2000 μ s^−1^

### Growth test of Bifidobacteria and Lactobacillales with AOS

The growth of two Bifidobacteria species, *B. longum* and *B. adolescentis*, was evaluated under conditions similar to those used for *R. gnavus*. AOS concentrations of 0, 0.1, 0.2, 0.3 and 0.4% were tested, with six replicates (*N* = 6). For the culture of *B. adolescentis*, the glucose concentration in the RF medium was increased to 1.0%, and the incubation period was extended to 48 h to account for its slower growth rate. Similarly, the growth of two lactic acid bacteria, *L. plantarum* and *L. casei*, was assessed using a comparable method. In this case, MRS broth was used instead of RF medium, with AOS concentrations of 0, 1.0, 2.0, 3.0 and 5.0%. For *L. casei*, the MRS broth also included 0.2% sucrose. Growth assays for these lactic acid bacteria were conducted with four replicates (*N* = 4). Statistical analyses were performed using the Kruskal–Wallis test for intragroup statistics followed by the *post-hoc* Dunn’s multiple comparisons test.

### Growth test of mixed culture of *R. gnavus* and *B. longum* with AOS

The growth of a mixed culture of *R. gnavus* and *B. longum* was tested in the same manner as *R. gnavus*. However, the AOS concentration of 0.1% was tested with *N*=8 and simultaneously inoculated *R. gnavus* (OD_660_=1.42) and *B. longum* (OD_660_=2.63) in a medium with volumes of 20 and 0 µl, 20 and 1 µl, 20 and 10 µl and 0 and 1 µl, respectively. Statistical analyses were performed using the Kruskal–Wallis test for intragroup statistics followed by the *post-hoc* Dunn’s multiple comparisons test.

### Quantitative PCR (qPCR) analysis of co-cultured strains

After measuring turbidity, the remaining mixed culture of *R. gnavus* and *B. longum* simultaneously inoculated at volumes of 20 and 1 µl was centrifuged at 4 000 r.p.m. for 10 min to collect cells, which were suspended in 0.5 ml of water. The samples were incubated at 70 °C for 10 min and disrupted using zirconia beads on a FastPrep FP100A instrument (MP Biomedicals, Santa Ana, CA, USA) at 4300 r.p.m. for 10 min. The supernatant was centrifuged at 15 000 r.p.m. for 1 min and used as a DNA solution (*N*=8). The numbers of *gyrB* genes of *R. gnavus* and 16S rRNA genes of *B. longum* in these DNA solutions were determined via qPCR using QuantStudio 3 (Thermo Fisher Scientific). The reaction solution was prepared for each template DNA using the PowerTrack SYBR Green Master Mix (Thermo Fisher Scientific) according to the manufacturer’s instructions. The primers used to amplify *gyrB* from *R. gnavus* and the 16S rRNA from *B. longum* are listed in Table 2 [11,22]. The amplification cycle protocol consisted of initial denaturation at 95 °C for 2 min, followed by 40 cycles of denaturation at 95 °C for 10 s, annealing at 55 °C for 15 s, extension at 72 °C for 15 s and final extension at 72 °C for 1 min. After amplification, a melting curve analysis was performed to confirm the specificity of the PCR products. The relative abundance (%) of *R. gnavus* cells in the culture medium was estimated from the number of *gyrB* from *R. gnavus* and the 16S rRNA gene from *B. longum* divided by 4. This is because *R. gnavus* has four 16S ribosomal RNA genes in its genome.

### *Invivo* examination of the effect of AOS on the gut microbiota using a mouse model

In this study, we used a model with Balb/c mice weighing 15–20 g, which were purchased from SLC Japan (Hamamatsu, Japan). Mice were randomly grouped using Excel, with three mice per cage. They were kept in cages lined with soft chips at 24 ± 2 °C, maintained on a 12 h light/dark cycle, with airflow and *ad libitum* access to food and water. The cages were placed randomly to avoid the influence of environmental factors on the responses of mice.

All mice were divided into a control group (Ctrl group; *N*=6) and AOS intervention group (AOS group; *N*=6). After 1 week of pre-rearing with AIN-93G semi-synthetic feed (CLEA Japan), experimental diets were administered from week 0. The control diet constituted AIN-93G semi-synthetic feed (CLEA Japan), whereas the composition of the diets for the experimental groups was based on AIN-93G with certain modifications. In the AOS group, 5% cellulose was replaced with 5% AOS (Ina Food Industry, Japan). After 4 weeks of breeding, the mice were anaesthetized using isoflurane inhalation for autopsy. Anaesthesia was confirmed by loss of stand-up reflex and pain reflexes that occur due to stimulation of toes, tail and ears with tweezers. Thereafter, cardiac puncture was performed (euthanasia). During the autopsy, caecal contents were collected for DNA preparation. Caecal contents were stored at −80 °C until further use.

All animal studies were conducted following the Animal Research: Reporting of In Vivo Experiments (ARRIVE) guidelines and the Animal Experimentation Guidelines of Nagoya University of Arts and Sciences. The study was approved by the ethics committee of the Laboratory Animal Care Committee of Nagoya University of Arts and Sciences in accordance with the standards of the Ministry of the Environment (approval No. 153).

### 16S rRNA gene sequencing

Caecal DNA extraction and 16S rRNA gene Next-Generation sequencing (NGS) were performed as shown previouslyhttps://link.springer.com/article/10.1186/s12866-023-03021-6 [14]. The data generated by the MiSeq sequencing system were processed, statistically analysed and visualized using the EzBioCloud 16S database and the 16S microbiome pipeline provided by ChunLab Inc. (EzBioCloud 16S-based MTP app, available at https://www.EZbiocloud.net). Analysis of alpha diversity indices, including Chao1 and Shannon was performed using the Mann–Whitney test. The Wilcoxon rank-sum test and permutational multivariate ANOVA (PERMANOVA) with 9999 permutations, respectively, were used to test for differences in alpha and beta diversity between the groups. Beta diversity was assessed through distances calculated using the Jenson–Shannon divergence. Potential taxonomic biomarkers distinguishing each group were identified using the linear discriminant analysis effect size (LEfSe) algorithm at the family and genus level, following the default parameters. In the EzBioCloud’s LEfSe analysis, the ‘*P*-value (FDR)’ is effectively used as the *Q*-value, and is thus referred to as the ‘*Q*-value’.

### Quantitative PCR analysis of *nan* levels

The abundance of the *nan* gene (*nan* levels) was quantified using qPCR with the QuantStudio 3, following a previously reported protocol [14]. In brief, the reaction mixture for each template DNA was prepared using PowerTrack SYBR Green Master Mix according to the manufacturer’s instructions. The *nan* primer set shown in Table 1 was employed, targeting conserved regions of *nanA* in *R. gnavus* and closely related Lachnospiraceae strains. The forward primer, nanA_113F, binds at positions 61–77 bp, while the reverse primer, nanA_113R, binds at positions 152–173 bp of the *nanA* gene in *R. gnavus* ATCC 29149. The amplification cycle protocol comprised initial denaturation at 95 °C for 2 min, followed by 40 cycles of denaturation at 95 °C for 10 s, annealing at 50 °C for 15 s, extension at 72 °C for 20 s and final extension at 72 °C for 1 min. Primers F_Bact 1369 and R_Prok1492 shown in Table 1 were used to amplify the total 16S rRNA genes as previously described [30], and qPCR was performed in the same manner as described above, except with an annealing temperature of 60 °C and extension for 15 s. The *nan* level in the DNA sample was calculated by dividing the number of *nanA* homologue genes by the total number of 16S rRNA genes, followed by an adjustment through multiplication by 4. This adjustment accounts for the variability in the number of 16S rRNA genes in bacteria, which can range from 1 to 15 copies per genome [31], with an assumed average of approximately four 16S rRNA genes per bacterium. Statistical analyses of qPCR were performed using the Mann–Whitney test.

### Statistical analysis

Statistical analyses were performed using GraphPad Prism 10.3 (GraphPad Software Inc., San Diego, CA, USA), following the methods outlined in each section. Statistical significance was defined as *P*<0.05, with thresholds for significance indicated as follows: *P<0.05, **P<0.01, ***P<0.001, ****P<0.0001.

## Results

### Inhibitory effect of AOS on the growth of *R. gnavus* and *F. nucleatum*

**Fig. 1.**
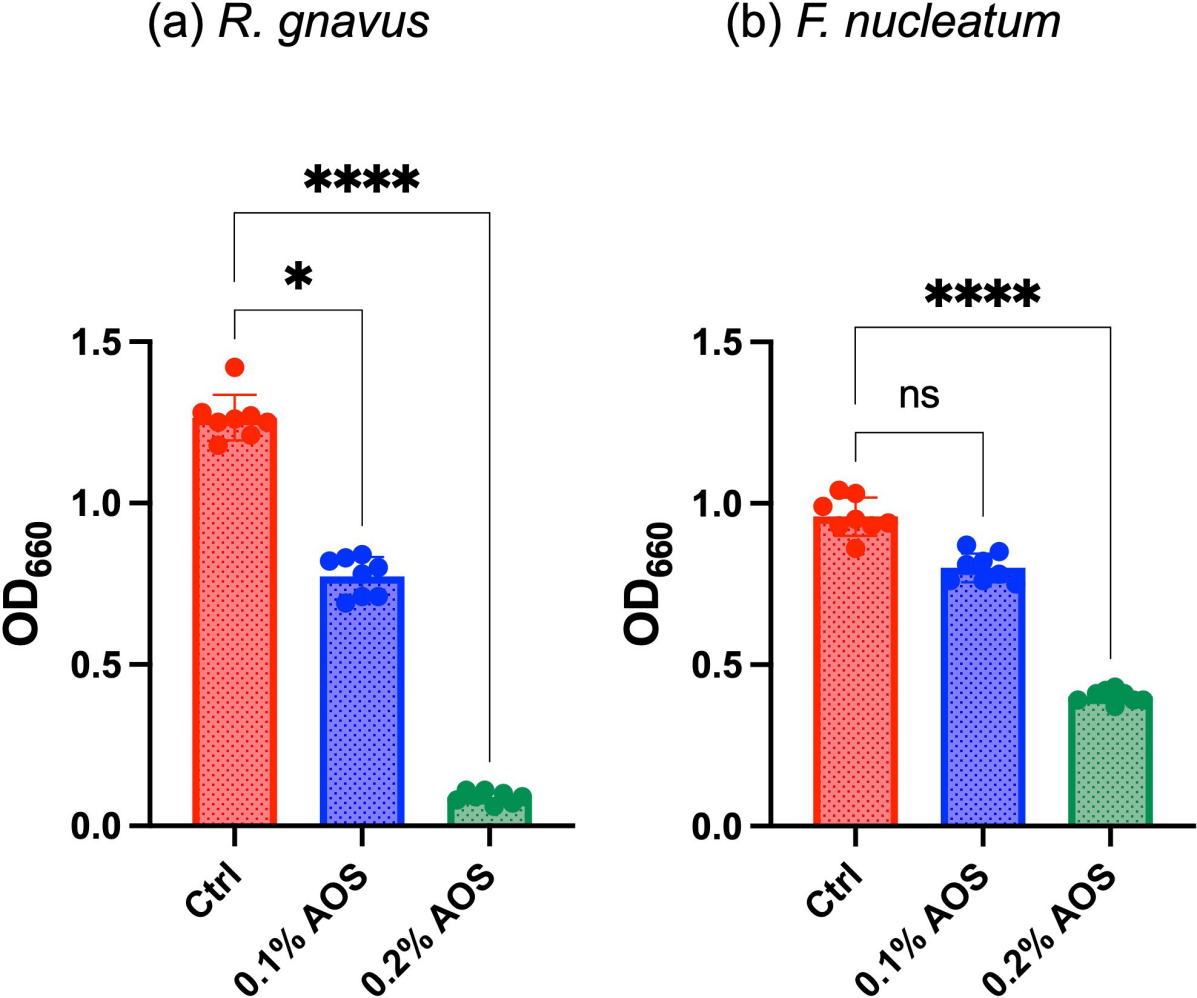
Turbidity (OD_660_) of (**a**) *Ruminococcus gnavus* after 23 h of incubation and (**b**) *Fusobacterium nucleatum* after 9 h of incubation in medium containing agarooligosaccharides at 0% (Ctrl), 0.1% (0.1% AOS) or 0.2% (0.2% AOS) concentrations (*N*=8). Each circle represents an individual culture, and bars indicate mean ± sd. Statistical significance is denoted as follows: *P<0.05, ****P<0.0001, ns: not significant.

The growth-inhibitory effects of AOS were clearly observed in cultures of *R. gnavus* and *F. nucleatum*. Growth assays showed a significant reduction in turbidity (OD_660_) in *R. gnavus* cultures at AOS concentrations of 0.1 and 0.2% compared to the control after 23 h of incubation (Fig. 1a). Similarly, AOS demonstrated inhibitory effects on *F. nucleatum*, with a significant decrease in turbidity at a 0.2% concentration after just 9 h of incubation (Fig. 1b).

### Inhibitory effect of AOS constituent sugars on the growth of *R. gnavus* and *F. nucleatum*

**Fig. 2.**
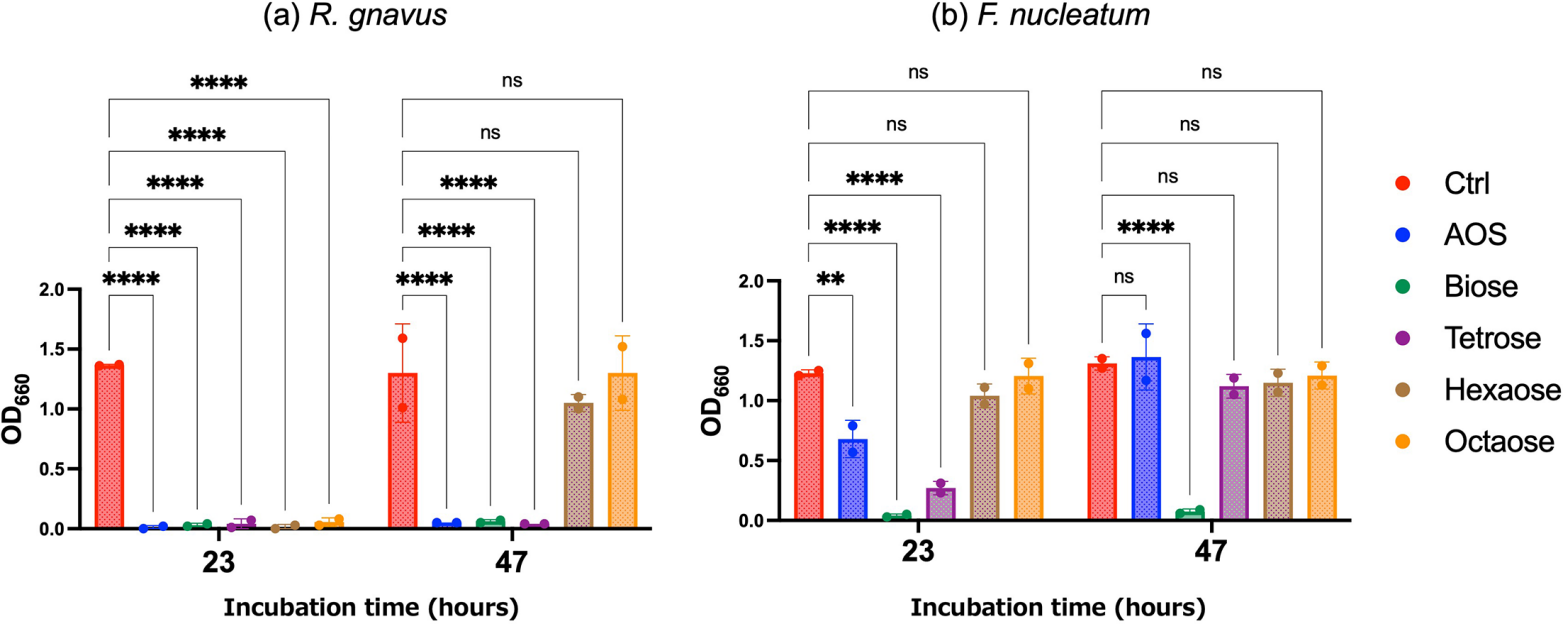
Turbidity (OD_660_) of (**a**) *Ruminococcus gnavus* and (**b**) *Fusobacterium nucleatum* in medium without agarooligosaccharides (Ctrl) and with 0.1% agarooligosaccharides (AOS) or its constituent sugars (biose, tetraose, hexaose or octaose) after incubation for 23 or 47 h (*N* = 2). Each circle represents an individual culture, and bars indicate mean±sd. Statistical significance is denoted as follows: **P<0.01, ****P<0.0001, ns: not significant.

The growth-inhibitory effects of AOS constituent sugars were evaluated in cultures of *R. gnavus* and *F. nucleatum*. Turbidity (OD_660_) in *R. gnavus* cultures was significantly reduced by the addition of AOS or its constituent sugars (biose, tetraose, hexaose and octaose) at a concentration of 0.1% (*w*/*w*) after 23 h of incubation. However, hexaose and octaose did not maintain their inhibitory effects after 47 h (Fig. 2a). In contrast, the growth of *F. nucleatum* was significantly inhibited by AOS, biose or tetraose after 23 h, with biose being the only compound to sustain significant inhibitory activity after 47 h of incubation (Fig. 2b).

### Comprehensive gene expression analysis of AOS-treated *R. gnavus*

**Fig. 3.**
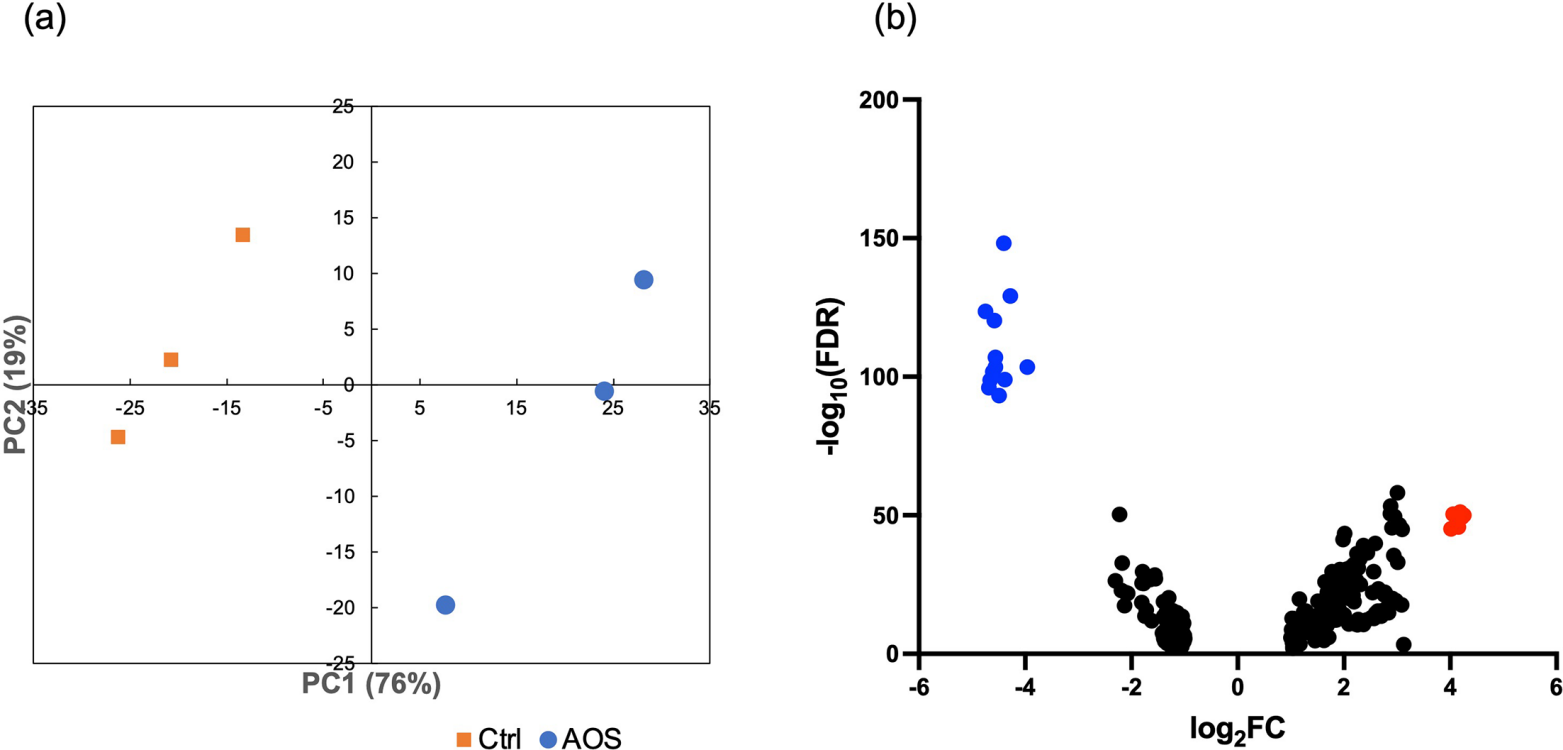
RNA-seq analysis of *Ruminococcus gnavus* in the presence and absence of 0.1% agarooligosaccharides (AOS) and verification by iDEP.96 (*N*=3). (**a**) The PCA plot illustrates clustering of RNA-seq samples treated with and without AOS. (**b**) The volcano plot visualizes the RNA-seq data, with blue dots representing most repressed genes and red dots indicating most induced genes.

To examine the mechanism underlying the inhibitory effect of AOS on the growth of *R. gnavus*, a comprehensive gene expression analysis using RNA-seq was performed on *R. gnavus* cultured in the presence or absence of AOS. To avoid complete suppression of cellular metabolism, a weak inhibitory concentration (0.1% [*w*/*w*]) was selected. After filtering the raw reads, 19,673,212, 19,502,998 and 19,360,508 clean transcriptome reads were found in the control samples, whereas 21,307,158, 19,330,559 and 14,153,917 clean reads were obtained from the AOS-treated samples. A complete list of all the reads is provided in Supplementary File 1, available in the online version of this article. Among these genes, 255 differentially expressed genes were identified using the calculated gene expression levels (|log_2_FC (fold change)| > 1, FDR < 0.05), with 151 and 104 genes markedly up- and downregulated in *R. gnavus* following AOS treatment, respectively. The principal component analysis (PCA) plot illustrates a distinct separation between the control and AOS-treated groups, with the first principal component (PC1) explaining 76% of the variance (Fig. 3a). Additionally, a volcano plot highlights significantly differentially expressed genes that AOS downregulated (Fig. 3b).

### Up- and downregulated genes in response to AOS in *R. gnavus*

Protein annotation with *Z*-score (PANNZER) analysis was employed to predict the functional descriptions and GO classifications of proteins affected by AOS treatment. Genes significantly up- or downregulated by AOS (|log2FC| > 3) are listed in Table 3. The gene cluster most highly induced by AOS, including RS16950, RS16945, RS16940, RS16955, RS16965 and RS16960 [upregulated gene group (URGs), marked with red dots in Fig. 3b], forms a single operon associated with the catabolism of carbon sources. Conversely, the most repressed genes by AOS form another operon, comprising RS03245, RS03220, RS03210, RS03240, RS03230, RS03215, RS03235, RS03225, RS03200, RS03205, RS03250 and RS03195 (downregulated gene group marked with blue dots in Fig. 3b), which saw a 4.0- to 4.8-fold reduction in expression. This cluster includes the *fab* genes responsible for encoding proteins involved in fatty acid biosynthesis.

**Table 3.** Upregulated genes (top table) and downregulated genes (bottom table) by agarooligosaccharides (|log2FC| > 3)

**Gene_id**	**Log**_**2**_ **FC**	**Padj_AO/Control**	**Gene product**	**Gene name**	**Description (shown by Pannzer2)**		**Biological process (shown by Pannzer2)**		
					**Estimated PPV (>0.5)**	**Description**	**Estimated PPV (>0.5)**	**GO-id**	**Description**
FXV78_RS16950	4.354472781	0.009953477	hypothetical protein	-	0.53	Peptidase	-	-	-
FXV78_RS16940	4.312877454	0.010057642	YhcH/YjgK/YiaL family protein	tabA	0.65	DUF386 domain-containing protein	-	-	-
FXV78_RS16955	4.269078849	0.012166236	mannonate dehydratase	uxuA	0.71	Mannonate dehydratase	0.8	GO:0006064	glucuronate catabolic process
FXV78_RS16945	4.214671917	0.008666144	hydantoinase	-	0.54	Hydantoinase /oxoprolinase	-	-	-
FXV78_RS16965	4.178587306	0.008902662	hypothetical protein	-	-	-	-	-	-
FXV78_RS16960	4.144592007	0.011805824	3-dehydro-l-gulonate 2-dehydrogenase	yiaK	0.81	3-dehydro-l-gulonate 2-dehydrogenase	-	-	-
FXV78_RS11805	3.304991514	0.010308326	alpha-galactosidase	-	-	-	0.67	GO:0016052	carbohydrate catabolic process
FXV78_RS14030	3.228410093	0.009050114	alpha-galactosidase	-	-	-	0.67	GO:0016052	carbohydrate catabolic process
FXV78_RS16625	3.208232713	0.007294465	sugar ABC transporter permease	-	-	-	0.55	GO:0055085	trans membrane transport
FXV78_RS07385	3.172627054	0.014125052	bacteriophage Gp15 family protein	-	-	-	-	-	-
FXV78_RS11785	3.146209795	0.007705183	hypothetical protein	-	0.64	WxL domain-containing protein	-	-	-
FXV78_RS11810	3.143038663	0.009218514	carbohydrate ABC transporter permease	-	-	-	0.55	GO:0055085	trans membrane transport
FXV78_RS14025	3.112073879	0.012210276	phosphopyruvate hydratase	eno	0.68	Enolase	0.7	GO:0006096	glycolytic process
FXV78_RS11790	3.111310165	0.007294465	extracellular solute-binding protein	-	-	-	0.55	GO:0055085	trans membrane transport
FXV78_RS11800	3.109170332	0.00719394	ABC transporter ATP-binding protein/permease	irtA	-	-	0.55	GO:0055085	trans membrane transport
FXV78_RS11060	3.038357162	0.033489332	hypothetical protein	-	0.73	Hemolysin XhlA	-	-	-
**Gene_id**	**Log**_**2**_ **FC**	**padj_AO/Control**	**Gene product**	**Gene name**	**Description (shown by Pannzer2)**		**Biological process (shown by Pannzer2)**		
					**Estimated PPV (>0.5)**	**Description**	**Estimated PPV (>0.5)**	**GO-id**	**Description**
FXV78_RS03245	−4.583053546	0.001443982	acyl carrier protein	acpP	0.51	Acyl carrier protein	0.69	GO:0006633	fatty acid biosynthetic process
FXV78_RS03220	−4.50020503	0.001920424	acetyl-CoA carboxylase biotin carboxyl carrier protein	accB	0.56	Biotin carboxyl carrierprotein of acetyl-CoA carboxylase	0.69	GO:0006633	fatty acid biosynthetic process
FXV78_RS03210	−4.449626914	0.001729883	acetyl-CoA carboxylase biotin carboxylase subunit	accC	0.57	Biotin carboxylase	0.76 0.69	GO:2 001 295 GO:0006633	malonyl-CoA biosynthetic process fatty acid biosyntheticprocess
FXV78_RS03240	−4.413212202	0.001443982	enoyl-[acyl-carrier-protein] reductase FabK	fabK	0.78	Enoyl-[acyl-carrier-protein]reductase FabK	-	-	-
FXV78_RS03230	−4.372989501	0.001443982	3-oxoacyl-[acyl-carrier-protein] reductase	fabG	0.62	3-oxoacyl-[acyl-carrier-protein] reductase	0.74 0.69	GO:0008202 GO:0006633	steroid metabolic process fatty acid biosynthetic process
FXV78_RS03215	−4.368364331	0.001443982	ACP S-malonyltransferase	fabD	0.57	Malonyl CoA-acyl carrierprotein transacylase	-	-	-
FXV78_RS03235	−4.366262871	0.001806912	3-hydroxyacyl-ACP dehydratase FabZ	fabZ	0.63	3-hydroxyacyl-[acyl-carrier-protein] dehydratase FabZ	0.74 0.69	GO:0009245 GO:0006633	lipid A biosynthetic process fatty acid biosynthetic process
FXV78_RS03225	−4.301519283	0.001806912	beta-ketoacyl-ACP synthase II	fabF	0.6	3-oxoacyl-[acyl-carrier-protein] synthase 2	0.69	GO:0006633	fatty acid biosynthetic process
FXV78_RS03200	−4.198918304	0.001443982	nitronate monooxygenase family protein		0.52	Nitronate monooxygenase	-	-	-
FXV78_RS03205	−4.185994714	0.001806912	acetyl-CoA carboxylase carboxyl transferase subunit	accD	0.57	Multifunctional fusion protein	0.76 0.69	GO:2 001 295 GO:0006633	malonyl-CoA biosynthetic process fatty acid biosynthetic process
FXV78_RS03250	−4.08232655	0.001806912	ketoacyl-ACP synthase III	fabH	0.58	Beta-ketoacyl-[acyl-carrier-protein] synthase III	0.69	GO:0006633	fatty acid biosynthetic process
FXV78_RS03195	−3.756921586	0.001443982	MarR family winged helix-turn-helix transcriptional regulator	-	-	-	0.58	GO:0006355	regulation of DNA-templated transcription

### RT-qPCR analysis of *fab* genes in response to AOS in *R. gnavus* and *F. nucleatum*

**Fig. 4.**
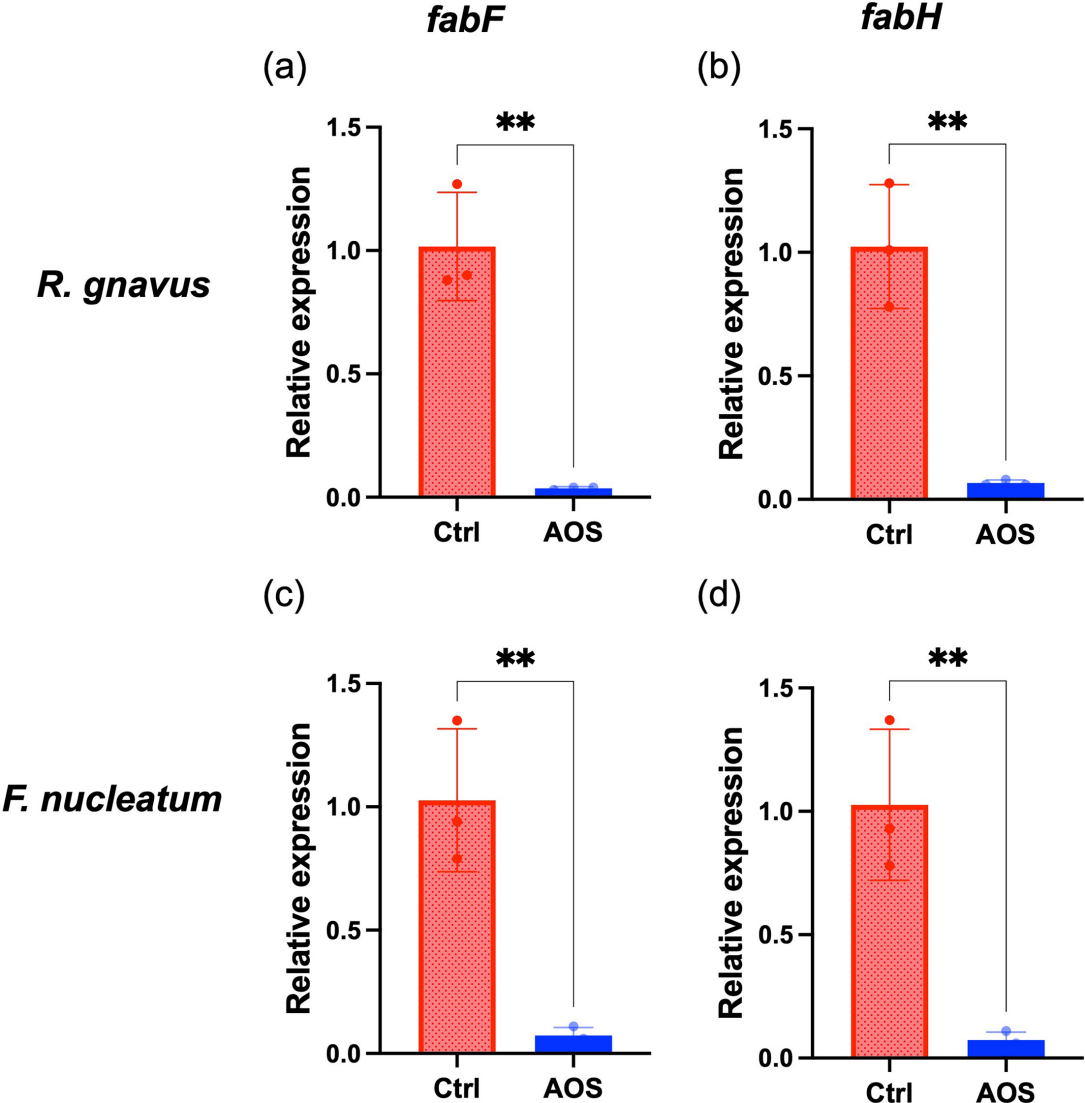
Differential gene expression analysis of *fab* genes by RT-qPCR. The expression levels of *fabF* and *fabH* in *Ruminococcus gnavus* and *Fusobacterium nucleatum* were examined by RT-qPCR in medium without agarooligosaccharides (Ctrl) and with 0.1% agarooligosaccharides (AOS) (*N* = 3). Each circle represents an individual culture, and bars indicate mean±sd. Statistical significance is denoted as follows: **P<0.01.

We assessed the effect of adding 0.1% (*w*/*w*) AOS to the culture medium on the expression levels of *fab* genes in both the Gram-positive bacterium *R. gnavus* and the Gram-negative bacterium *F. nucleatum*. RT-qPCR analysis using specific primers for *fabF* and *fabH* confirmed the downregulation of these genes in *R. gnavus* in the presence of AOS (Fig. 4a, b). Although the *fab* genes in *F. nucleatum* have not been well characterized, our genomic analysis identified an operon encoding homologues of FabH, FabD, AccP and FabF in its genome (GenBank: AE009951.2), including FN0148 (*fabH*), FN0149, FN0150 and FN0151 (*fabF*). RT-qPCR analysis showed that *fabF* and *fabH* were also downregulated in *F. nucleatum* following AOS treatment (Fig. 4c, d).

### AOS-induced changes in fatty acid composition in *R. gnavus* membrane

**Fig. 5.**
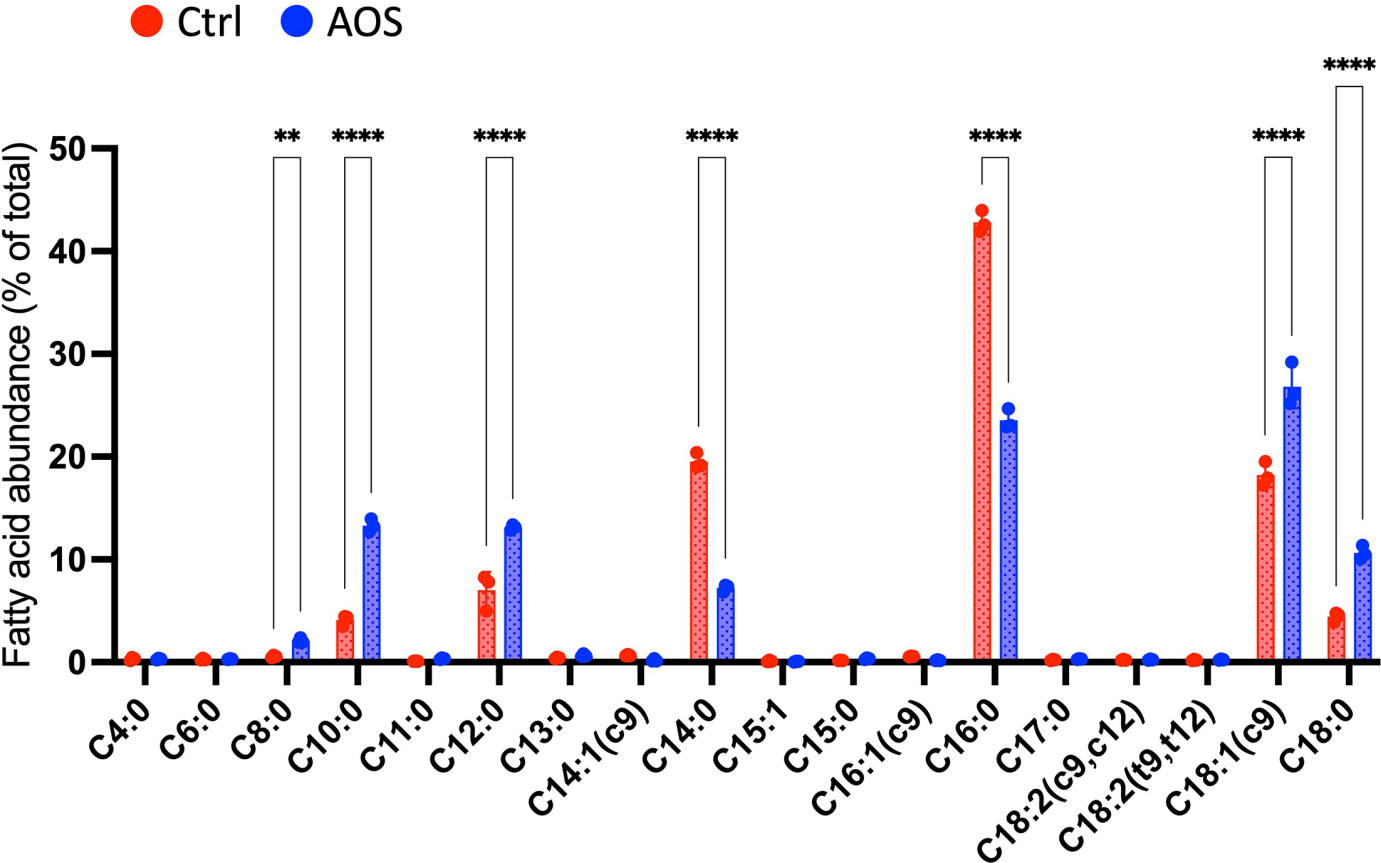
Fatty acid composition of *Ruminococcus gnavus* membranes in media without (Ctrl) and with 0.1% agarooligosaccharides (AOS) (*N*=3). Each circle represents an individual culture, and bars indicate mean±sd. Statistical significance is denoted as follows: **P<0.01, ****P<0.0001.

Given that AOS suppresses the expression of *fab* genes involved in fatty acid biosynthesis, we investigated the impact of 0.1% (*w*/*w*) AOS treatment on the fatty acid composition of *R. gnavus* cell membranes. This analysis showed that AOS treatment resulted in significant increases in certain medium-chain saturated fatty acids (C8, C10) and increases in C18 fatty acids at the expense of other long-chain fatty acids (C14, C16) (Fig. 5).

### Effect of AOS on the growth of Bifidobacteria and Lactobacillales

**Fig. 6.**
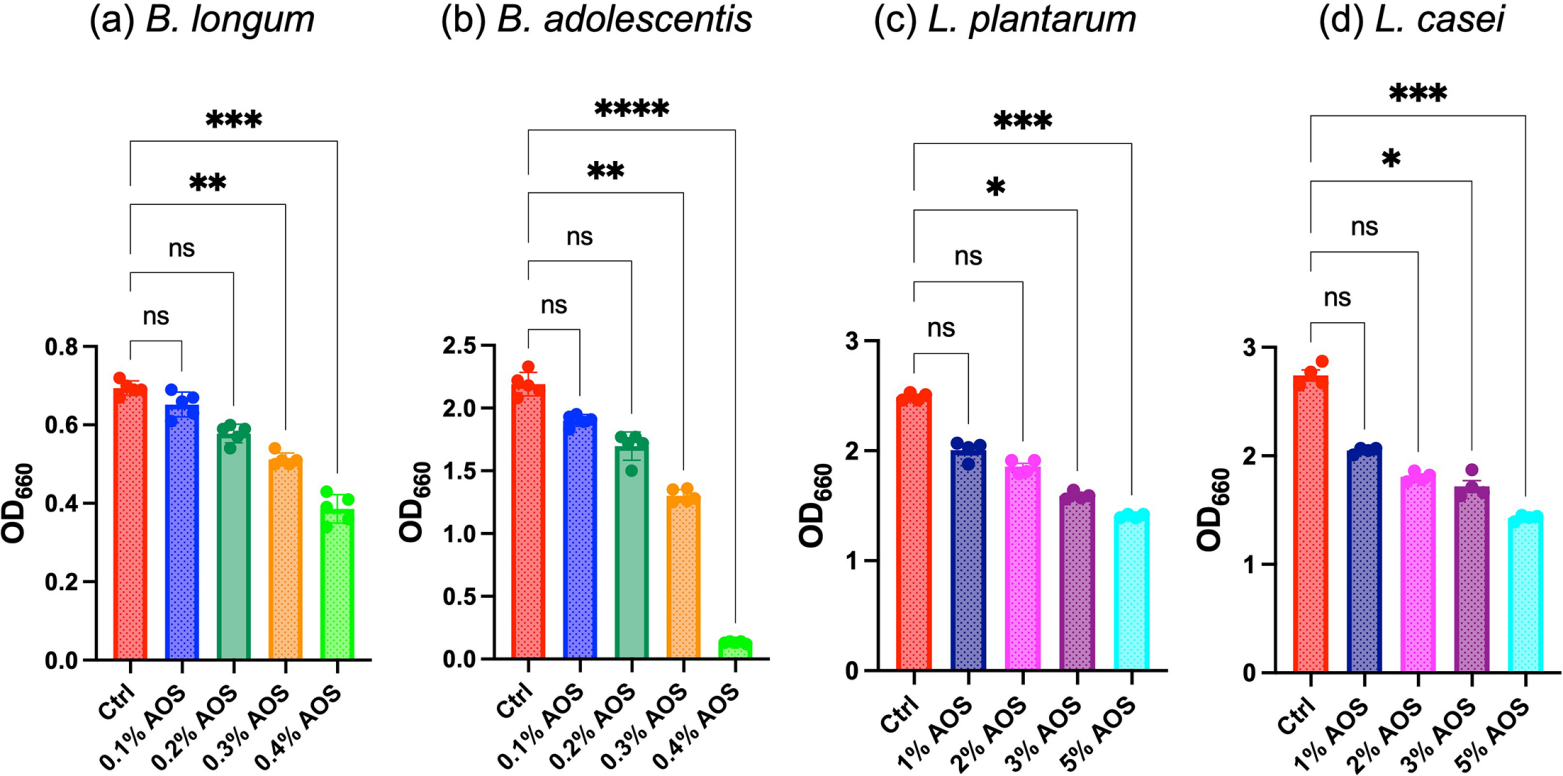
Turbidity (OD_660_) of (**a**) *Bifidobacterium longum* and (**b**) *Bifidobacterium adolescentis* after 23 and 48 h of incubation, respectively, in medium containing agarooligosaccharides (AOS) at concentrations of 0% (Control), 0.1% (0.1% AOS), 0.2% (0.2% AOS), 0.3% (0.3% AOS) and 0.4% (0.4% AOS) (*N*=6). Turbidity (OD_660_) of (**c**) *Lactiplantibacillus plantarum* and (**d**) *Lacticaseibacillus casei* after 23 h of incubation in medium containing AOS at concentrations of 1.0% (1% AOS), 2.0% (2% AOS), 3.0% (3% AOS) and 5.0% (5% AOS) (*N*=4). Each data point represents an individual culture, and bars indicate mean±sd. Statistical significance is denoted as follows: *P<0.05, **P<0.01, ***P<0.001, ****P<0.0001, ns: not significant.

The growth-inhibitory effects of AOS were observed in cultures of Bifidobacteria and Lactobacillales strains. For two Bifidobacteria species, *B. longum* and *B. adolescentis*, there were no significant differences in turbidity (OD_660_) compared to the control at AOS concentrations of 0.1 and 0.2%. However, at concentrations of 0.3% and higher, turbidity was significantly reduced relative to the control (Fig. 6a, b). A similar pattern was seen in two Lactobacillales species, *L. plantarum* and *L. casei*, where no significant differences in turbidity were observed at AOS concentrations of 1 and 2%. At concentrations of 3% and above, however, turbidity was significantly lower than in the control (Fig. 6c, d).

**Fig. 7.**
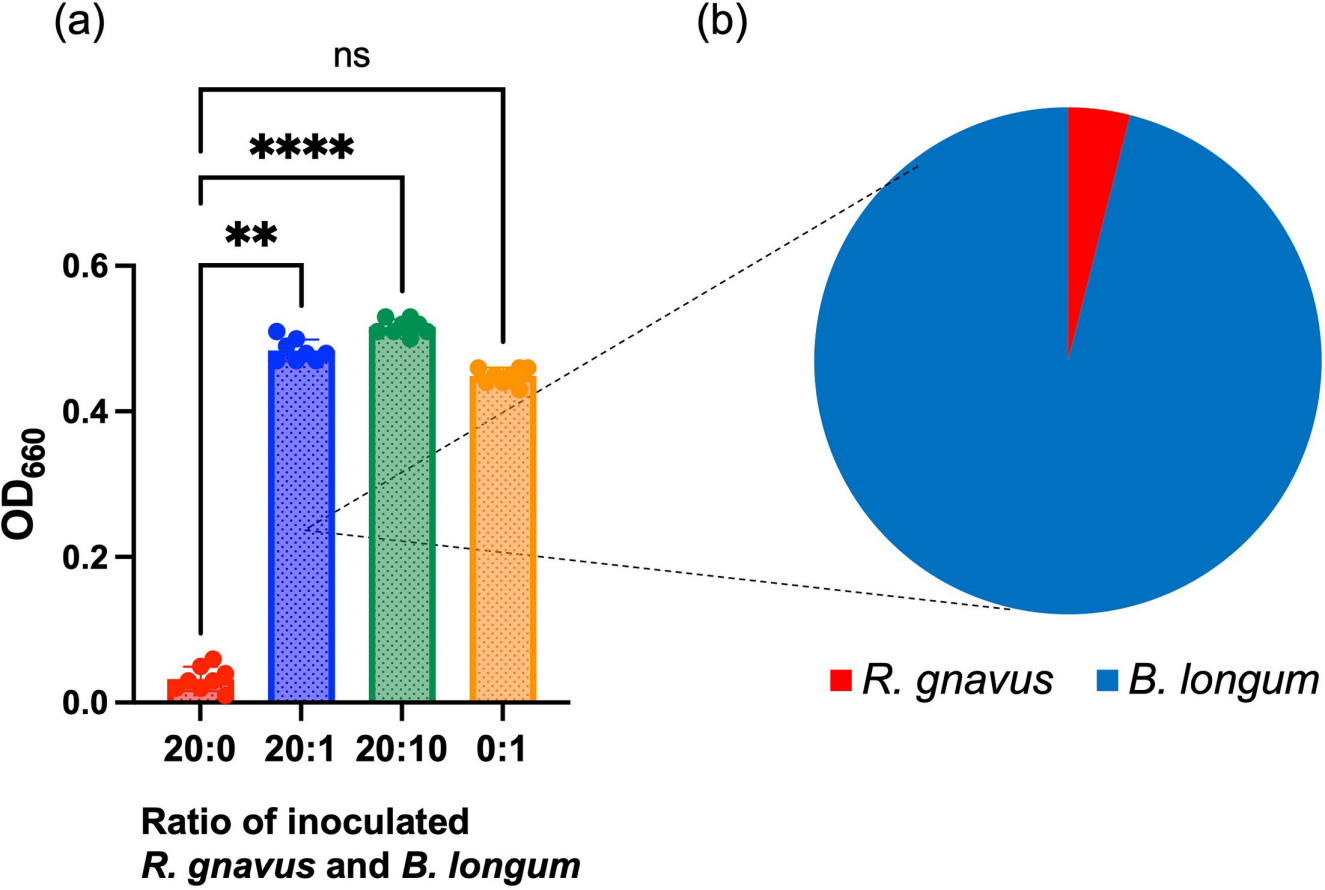
(**a**) Turbidity (OD_660_) of mixed cultures inoculated with *Ruminococcus gnavus* and *Bifidobacterium longum* at volume ratios of 20 : 1 and 20 : 10 and of cultures of the respective bacteria alone (20 : 0 for *R. gnavus* and 0 : 10 for *B. longum*), grown in medium containing 0.2% agarooligosaccharides for 23 h (*N* = 8). (**b**) Relative abundance (%) of *R. gnavus* and *B. longum* in the mixed culture (20 : 1 ratio) at the end of incubation assessed by qPCR. Circles represent individual cultures, and bars indicate mean±sd. Statistical significance is denoted as follows: **P < 0.01, ****P < 0.0001, ns: not significant.

Additionally, a mixed culture experiment was performed using *R. gnavus* and *B. longum* in a medium supplemented with 0.2% AOS. Mixed cultures with volume ratios of *R. gnavus* to *B. longum* at 20 : 1 and 20 : 10 showed significantly higher growth rates compared to cultures inoculated solely with *R. gnavus* and were comparable to those inoculated with only *B. longum* (Fig. 7a). In the specific mixed culture at a 20 : 1 ratio, qPCR analysis revealed that *B. longum* dominated the bacterial population, comprising over 96% of the total bacteria after incubation (Fig. 7b).

### Effect of AOS on mice gut microbiota

**Fig. 8.**
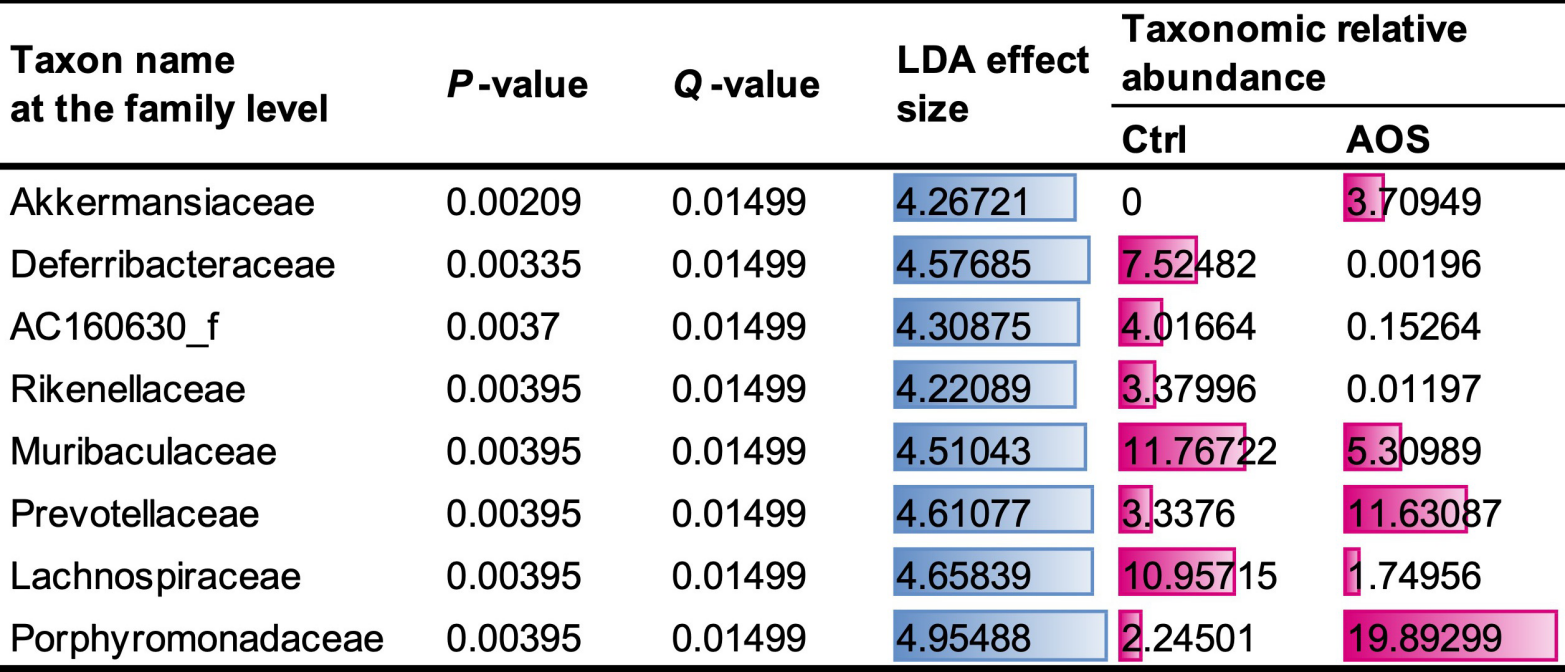
Results of the linear discriminant analysis (LDA) effect size analysis at the family level for distinguishing agarooligosaccharides intervention (AOS) group from the control (Ctrl) group, using criteria of *P*-value <0.001 and |LDA effect size| >4.0. The blue and red bars represent the relative quantities of the LDA effect size and taxonomic relative abundance, respectively.

**Fig. 9.**
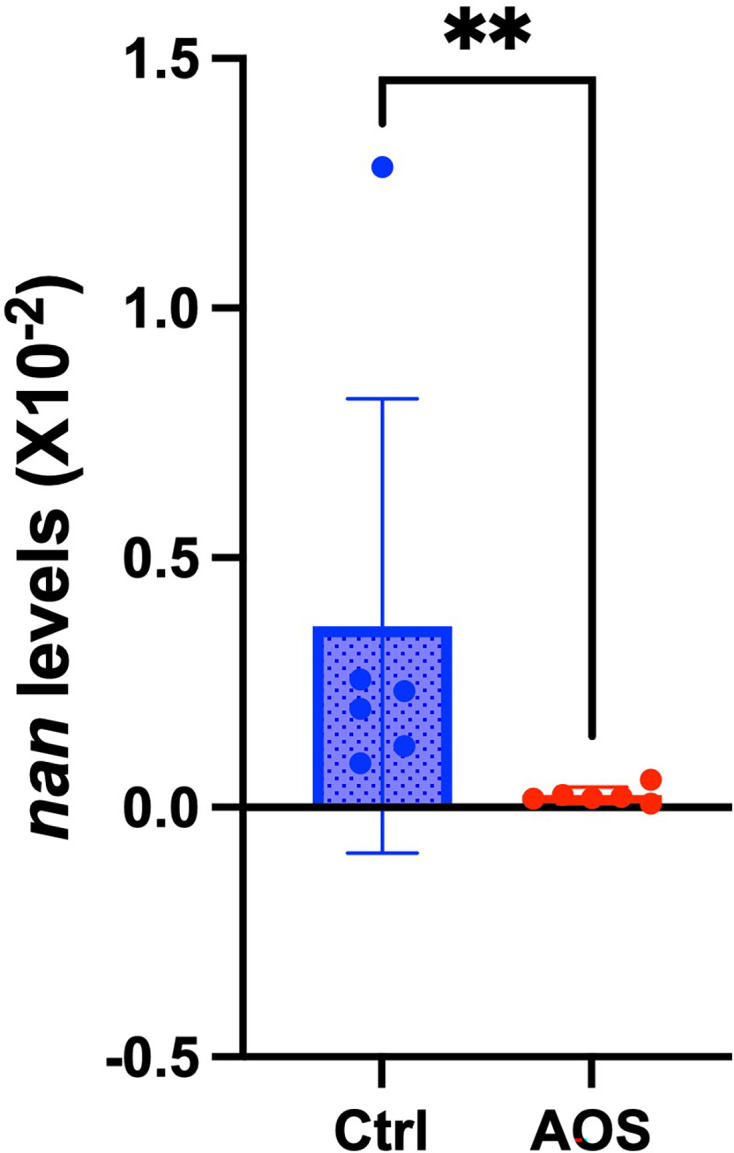
Quantitative PCR analysis of the abundances of *nan* gene (*nan* levels). The *nan* levels between the control (Ctrl) and the agarooligosaccharides intervention (AOS) groups are compared. Plots represent individual mice, and bars represent the mean ± sd. **P<0.01.

The impact of AOS on the gut microbiota of mice was analysed using DNA samples sequenced via 16S rRNA gene NGS, yielding an average of 24,130 ± 1,272 reads for the control group and 25,689 ± 1,299 reads for the AOS group (mean±se). Comparative analyses of caecal microbiota composition between the control and AOS groups at the family, genus and species levels are detailed in Supplementary File 2. Differences in microbiome diversity indices revealed significantly lower alpha diversity in the AOS group, as indicated by the Chao1 (*P*=0.006) and Shannon indices (*P*=0.004). Principal coordinate analysis plots demonstrated distinct separation between the groups (Supplementary File 3), and PERMANOVA analysis confirmed significant differences in beta diversity (*P*=0.005). Neither *R. gnavus* nor *F. nucleatum* were detected at abundances of at least 0.01% (Supplementary File 4). However, LEfSe analysis revealed that a significant change was observed in the Lachnospiraceae family, to which *R. gnavus* belongs, with a lower abundance in the AOS group (Fig. 8). Additionally, all 19 genera within the Lachnospiraceae family that showed substantial changes (*P*<0.01) were less abundant in the AOS group compared to the control group (Supplementary File 5). Conversely, the families Akkermansiaceae, Prevotellaceae and Porphyromonadaceae showed increased abundances in the AOS group (Fig. 8). qPCR using the *nan* primer set, which estimates the abundance of *nan* gene derived from *R. gnavus* and related Lachnospiraceae species as demonstrated in our previous study [14], was performed on caecal DNA samples to compare *nan* levels between the control and AOS groups. The AOS group showed significantly lower *nan* levels compared to the control group (*P* = 0.0022), as illustrated in Fig. 9.

## Discussion

In this study, AOS inhibited the growth of *R. gnavus* and *F. nucleatum* in a dose-dependent manner. A comparison of the growth inhibitory effects of AOS constituent sugars (biose, tetraose, hexaose and octaose) revealed that shorter DP sugars tended to be more effective in inhibiting growth. A previous study showed that AHG and AOS carrying AHG at the reducing ends showed growth-inhibitory activity against *S. mutans*, whereas NAOS with d-galactose at the reducing ends did not [8,32]. In this study, each constituent sugar of AOS was added to the culture medium at the same weight percentage (0.1%). Consequently, the molar abundance of AOS sugars featuring an AHG at the reducing end was higher in shorter DP sugars. This likely enhanced the inhibitory activity of the shorter DP sugars against *R. gnavus* and *F. nucleatum*. The fatty acid biosynthesis gene cluster (*fab* genes) was significantly downregulated in *R. gnavus* in the presence of AOS, with a decrease ranging from 4.0- to 4.8-fold. A similar effect was observed in *F. nucleatum*, where *fabF* and *fabH* were also downregulated following AOS exposure. The bacterial fatty acid synthesis pathway is crucial for cell membrane formation, and several studies have shown that fatty acid synthesis inhibitors exhibit potent bacteriostatic agent effects against both Gram-positive and Gram-negative bacteria [33]. This suggests that the inhibition of *fab* gene expression could be one of the mechanisms through which AOS exerts its growth-inhibitory effects.

Analysis of the fatty acid composition of *R. gnavus* membranes revealed significant changes: AOS treatment resulted in significant increases in certain medium-chain saturated fatty acids and increases in C18 fatty acids at the expense of other long-chain fatty acids. These changes suggest that the growth inhibitory effects of AOS may stem from its modulation of fatty acid biosynthesis genes in both *R. gnavus* and *F. nucleatum*. In comparative studies, long-chain polyunsaturated fatty acids, such as arachidonic acid (AA), which are rarely found in bacterial organisms, have been identified as having growth-inhibiting properties. Specifically, AA has been shown to inhibit the growth of *Streptococcus pneumoniae* by integrating into and disrupting the bacterial membrane due to its unique chain length and double-bond structure [34]. This integration leads to a dramatic downregulation of *fab* genes, similar to the effects observed with AOS treatment in *R. gnavus* and *F. nucleatum*. Furthermore, bacteriostatic agents like penicillin operate by disrupting bacterial growth through interference with cell wall synthesis, specifically binding to transpeptidases to prevent the cross-linking of peptidoglycan strands. Rogers *et al*. [35] reported that penicillin exposure results in the downregulation of fatty acid biosynthesis genes in *S. pneumoniae*, potentially altering the cell membrane to enhance bacterial survival in its presence. Moreover, modifications to other membrane components, such as phospholipids, have been associated with altered susceptibility to different classes of bacteriostatic agents, including macrolides [36]. Additionally, exposure to paracetamol has been linked to significant downregulation of *fab* genes in *S. pneumoniae*, as noted by Afzal *et al*. [37], indicating potential resistance mechanisms to this drug. In our study, the observed changes in the fatty acid composition of *R. gnavus* membranes may represent a resistance mechanism against the effects of AOS, similar to adaptations seen in response to other bacteriostatic agents.

Most AOS-induced genes, URGs, were present as a single operon. RS16940 encoded proteins annotated to mannonate dehydratase, and RS16965 encoded proteins annotated to 3-dehydro-l-guronic acid 2-dehydrogenase. These enzymes metabolize mannonate and 3-dehydro-l-guronic acid, respectively. Unlike most six-carbon sugars, which exist in the pyranose form in water, the unique bicyclic AHG has been reported to transition to the aldehyde form and exist in its hydrated form under aqueous conditions [32]. These results suggest that the induced mannonate dehydratase gene product and the 3-dehydro-l-guronic acid 2-dehydrogenase gene product barely contribute to the degradation of AOS and may represent a second AOS resistance mechanism.

In the current study, AOS selectively inhibited the growth of potentially disease-associated bacteria (*R. gnavus* and *F. nucleatum*) at concentrations that had relatively little inhibitory effect on beneficial bacteria such as *Bifidobacteria* and *Lactobacillales*. This suggests that an appropriate intervention with AOS could reduce populations of disease-associated bacteria while promoting the growth of beneficial bacteria, introducing a novel concept in prebiotics. These observations are supported by previous results from *in vitro* fermentation studies using human faecal inocula with AOS, where AOS increased concentrations of short-chain fatty acids and modulated the microbiota composition [7]. A similar pattern of selective bactericidal activity has been observed in Paneth cells of the small intestinal epithelium [38]. These cells secrete the antimicrobial peptide *α*-defensins in response to bacterial stimuli. The oxidized form of *α*-defensins demonstrates potent bactericidal effects against pathogenic bacteria, but spares beneficial bacteria such as Bifidobacteria and Lactobacillales, thereby regulating the intestinal microbiota through selective bactericidal action that supports host health.

The mechanism behind the selective growth-inhibitory effect of AOS remains unclear. However, Lactobacillales species, particularly *L. plantarum*, can efficiently utilize externally supplied fatty acids. In these bacteria, the presence of external fatty acids can completely suppress endogenous fatty acid synthesis, allowing them to construct phospholipids entirely from external sources [39]. Similarly, Bifidobacteria, though not as adept as Lactobacillales strains, can also utilize certain external fatty acids [40]. This ability might help these bacteria compensate for the significant downregulation of *fab* genes induced by AOS, thereby supporting their continued growth.

AOS has been reported to exhibit anti-inflammatory properties. *In vitro* studies have demonstrated that AOS modulates immune responses in both mouse macrophages and human monocytes [41], and its administration has been shown to inhibit trinitrobenzene sulfonic acid-induced colitis in mice [42]. Based on our findings, we propose two potential mechanisms for the anti-inflammatory effects of AOS. One involves the reduction of Lachnospiraceae strains, including *R. gnavus*, following AOS administration, which may contribute to the suppression of inflammation. Similar reductions in Lachnospiraceae abundance have also been observed in previous AOS intervention studies in mice [43]. However, this hypothesis requires careful consideration, as not all Lachnospiraceae are harmful. Some members, such as *Blautia*, are associated with health benefits and are currently being investigated as next-generation probiotics [44]. Additionally, certain Lachnospiraceae strains within *Clostridium* cluster XIVa are important producers of butyrate, a beneficial short-chain fatty acid essential for gut health [45]. On the other hand, various Lachnospiraceae strains possess homologues of the *nan* gene, which is involved in mucin degradation and has been associated with leaky gut and IBD [14]. Our qPCR analysis showed significantly lower *nan* levels in the AOS-treated group, suggesting that AOS reduces the abundance of potentially pathogenic *nan*-harbouring strains. This conclusion, based on the detection of a functional pathogenic gene rather than the 16S rRNA gene, confirms that AOS specifically reduces inflammatory *nan*-harbouring strains, thereby contributing to the suppression of inflammation.

Another potential mechanism is that AOS may influence inflammatory processes by affecting fatty acid metabolism in gut microbiota. Monounsaturated fatty acids like oleic acid have been reported to promote inflammatory diseases by altering T cell metabolism [46]. Takeuchi *et al*. further demonstrated that faecal-derived fatty acids impair intestinal tight junction integrity in mice [47]. Their study also found that monocolonization of mice with *Escherichia coli* overexpressing *fadR* – which increases *fab* gene expression – led to significantly higher expression of the *lbp* gene in perigonadal fat tissue, encoding lipopolysaccharide-binding protein, which triggers immune responses. These findings suggest that AOS may play a role in reducing inflammation by inhibiting *fab* gene expression in gut microbiota.

AOS administration has also been shown to reduce obesity in mice [43]. Furthermore, a study by Takeuchi *et al.* [47] revealed that overexpression of *fab* genes in the gut led to higher expression of the *lep* gene, which encodes leptin, in perigonadal fat tissue, accompanied by significant increases in body mass. Leptin, a hormone primarily produced by adipose tissue, plays a key role in regulating energy balance by inhibiting hunger, which in turn reduces fat storage in adipocytes. When leptin levels increase, it typically signals to the brain that the body has sufficient energy reserves, curbing appetite and promoting energy expenditure. However, chronic overexpression of leptin can lead to leptin resistance, a condition where the body no longer responds effectively to the hormone’s signals, often observed in obesity [48]. These findings suggest that AOS may contribute to obesity reduction by inhibiting *fab* gene expression in the gut microbiota. Additionally, AOS treatment in this study significantly increased the abundance of the Akkermansiaceae, Prevotellaceae and Porphyromonadaceae families, which have been linked to anti-obesity effects in several studies [49,51]. This suggests another potential mechanism through which AOS may influence the gut microbial composition and inhibit obesity in mice.

Recently, research has highlighted a selective control of bacterial growth using *β*-galactosides, specifically galactosyl-*β*-1,4-l-rhamnose (Gal-*β*1,4-Rha) [52]. In this study, it was observed that bacteria capable of utilizing Gal-*β*1,4-Rha as their sole carbon source thrived, whereas those unable to use this compound did not proliferate. In contrast, AOS present a novel approach to prebiotics. AOS exhibits an inhibitory effect on the growth of potentially disease-associated bacteria even in the presence of glucose as a carbon source. This unique characteristic suggests that AOS has the potential to be a promising candidate for advancing prebiotic development and enhancing gut health by selectively suppressing harmful bacterial populations. Moreover, AOS may be even more effective when combined with other prebiotics, such as 1-kestose, which has been shown to significantly increase the populations of beneficial microorganisms, including Bifidobacteria and Lactobacillales, in both *in vitro* and *in vivo* studies [53,55].

This study has several important limitations that should be addressed. First, the growth inhibitory effects of AOS on *R. gnavus* and *F. nucleatum* – both of which are known for their strain-specific roles in inducing gastrointestinal disorders – were assessed using only a single strain for each species. This limits the ability to generalize these findings across different strains of these bacteria, which may vary in their sensitivity to AOS. Similarly, the limited inhibitory effects of AOS on beneficial Bifidobacteria and Lactobacillales were evaluated based on only two strains from each species, which may not fully represent the diversity and behaviour of these bacterial groups in broader contexts. As a result, the generalizability of the findings across multiple strains and species remains uncertain. Additionally, while it has been hypothesized that the suppression of *fab* operon gene expression by AOS may be a key factor in the observed growth inhibition of gut bacteria, the underlying molecular mechanisms behind this suppression remain poorly understood. A more thorough exploration of how AOS interacts with bacterial gene regulation could provide critical insights into its mode of action. If these mechanisms are clarified, it would allow us to better predict the effects of AOS across different bacterial species, potentially overcoming the limitations posed by strain-specific variations. Moreover, in our *in vivo* studies, *R. gnavus* and *F. nucleatum* were not detectable in the gut microbiota of the mice used, preventing us from verifying the reduction in these bacterial populations as seen in the *in vitro* experiments. This raises questions about whether the findings from the *in vitro* setting translate accurately to *in vivo* conditions. To address this, future studies may need to employ more sophisticated models, such as human flora-associated mice, which better mimic the human gut environment, or human clinical trials that can provide direct evidence of AOS’s effects on these bacteria within the human gastrointestinal tract. These approaches would help to validate and extend the current findings, providing a more comprehensive understanding of AOS’s potential to introduce a novel concept in prebiotics.

## Conclusion

AOS demonstrated a novel concept of prebiotics by selectively inhibiting the growth of potentially disease-associated bacteria, such as *R. gnavus* and *F. nucleatum*, as well as downregulating the expression of fatty acid biosynthetic *fab* genes. Notably, these effects occurred at concentrations that did not affect the growth of beneficial bacteria like Bifidobacteria and Lactobacillales. *In vivo* studies with mice showed that AOS intervention significantly reduced the relative abundance of the Lachnospiraceae family, to which *R. gnavus* belongs, along with its potentially pathogenic *nan* gene.

## supplementary material

10.1099/mic.0.001510 Uncited Supplementary Material 1.

## Abbreviations

AA: arachidonic acid
AHG: *α*-1,3-linked-3,6-anhydro-l-galactose
AOS: agarooligosaccharides
cDNA: complementary DNA
DP: degree of polymerization
FC: fold change
FDR: false discovery rate
Gal-*β*1,4-Rha: galactosyl-*β*1,4-l-rhamnose
GC-MS: gas chromatography-mass spectrometry
GO: gene ontology
IBD: inflammatory bowel disease
LEfSe: linear discriminant analysis effect size
MRS: de Man, Rogosa and Sharpe
nan levels: abundance of the nan gene
NAOS: neoagarooligosaccharide
NGS: Next-Generation sequencing
PC: principal component
PERMANOVA: permutational multivariate ANOVA
qPCR: quantitative PCR
RF: Reinforced Flora growth
RNA-seq: RNA sequencing
RT-qPCR: quantitative reverse transcription PCR
